# An *in vitro* model of stiffened colonic mucosa exhibits altered epithelial behavior

**DOI:** 10.1088/1758-5090/ae2cf2

**Published:** 2025-12-29

**Authors:** Angelo Massaro, Cecilia Villegas Novoa, Nancy L Allbritton

**Affiliations:** Department of Bioengineering, University of Washington, Seattle, WA, United States of America

**Keywords:** microphysiologic systems, intestine, organ-on-chip, stiff matrix, colon crypts

## Abstract

Stiffening of the extracellular matrix underlying the epithelial cells of the large intestine is associated with aging as well as many diseases. Yet the impact of the stiffened matrix on epithelial physiology remains poorly understood. A 2D and 3D microphysiological model of the large intestine was developed using a collagen scaffold with a physiologic or excessive stiffness (Young’s moduli of 2.84 ± 0.85 kPa and 15.9 ± 0.73 kPa) by altering the collagen concentration within the substrate. Diffusion of a 10 and 40 kDa fluorescent dextran was significantly different between the physiologic and stiff scaffold (97.8 vs 79.8 *µ*m^2^ s^−1^ [10 kDa] and 68.2 vs 56.8 *µ*m^2^ s^−1^ [40 kDa], respectively). When primary human epithelial cells of the large intestine were grown as a 2D monolayer, cultures on the physiologic scaffold grew to a significantly higher density with more proliferative and fewer differentiated cells than cultures on the stiffened scaffold. Three-dimensional crypt arrays were also fabricated with the physiologic and stiff substrates, populated with cells, and a growth factor gradient applied. The cell density, proliferation, and height-to-width ratio was significantly greater for cells on the physiologic scaffold relative to that of cells on the stiffened scaffolds. Placement of a layer of intestinal fibroblasts below the epithelium on the crypt arrays did not mitigate the impact of the stiffened substrate. Bulk-RNA sequencing revealed 378 genes that were significantly upregulated and 385 genes significantly downregulated in the stiffened vs physiologic scaffolds. This work demonstrates that a molded collagen hydrogel can be used to mimic the biophysical characteristics of a stiffened intestinal stroma, recapitulating physiology observed *in vivo*. This *in vitro* model of polarized crypts with a tunable underlying substrate will enable an improved understanding of intestinal epithelial cell morphology, stem cell maintenance and lineage allocation within a stiffened environment.

## 1. Introduction

The large intestine, or colon, lies near the end of the gastrointestinal tract and is responsible for absorbing nutrients, storing waste, and housing the gut microbiome. Colonic tissue is organized concentrically with the innermost layer, the mucosa, consisting of epithelial cells above supportive stromal cells. The epithelium has secretory and absorptive functions, and, in healthy individuals, the layer is continuously regenerating from a source of proliferating, pluripotent stem cells found in the base of 500 *µ*m tall, 100 *µ*m wide invaginations called crypts. Underlying mesenchymal, immune, endothelial, and neural cells help support the epithelial layer to renew continuously and function as a preferential barrier, allowing in nutrients and water while keeping out foreign pathogens. Supporting fibroblasts play a role in shaping the physical microenvironment experienced by the epithelium by laying down and reshaping the extracellular matrix (ECM) [1]. The noncellular intestinal stroma is composed primarily of fibrous proteins including collagen, laminin, elastin, and fibronectin as well as non-fibrous glycoproteins [2]. Not surprisingly this matrix is highly deformable with a Young’s modulus of 2.9 kPa so that semi-solid waste materials can be propelled through the organ via waves of contraction directed by surrounding smooth muscle [3].

A stiffened matrix with a significantly increased Young’s modulus (16.7 kPa) can accompany gastrointestinal diseases such as colorectal cancer or inflammatory bowel disease [3]. The stiffened tissue is thought to be due to excessive ECM deposition including collagen accumulation. The decrease in stromal deformability is part of a larger pathophysiologic process termed fibrosis that can lead to bowel narrowing and dysfunction [4]. Strictures, or localized regions of stiffened colon tissue, are often accompanied by inflammation but the immune response and fibrotic stiffening can be an independent and self-perpetuating processes. Individuals with stiffened intestinal tissue experience a decrease in quality of life due to obstruction, nausea, vomiting, and abdominal pain as well as an increased risk of colon cancer as compared with healthy individuals [5–8]. A clinical treatment for intestinal stiffening or fibrosis does not yet exist, and a significant number of individuals with ongoing fibrosis will require surgery to remove sections of strictured intestine [9].

Beyond the colon, substrate stiffness is known to impact a diverse array of cellular properties including cytoskeletal structure, cell morphology, cell adhesion, and cell migration in many cell types [10–15]. Properties of the substrate on which a cell is attached affects cell fate and larger scale tissue dynamics as well [16, 17]. For epithelium specifically, increased stiffness may restrict stemness and encourage a secretory phenotype amongst differentiated cells [18, 19]. Changes in epithelial behavior can also persist after a cell returns to an environment with normal stiffness [20]. A high-throughput assay for evaluating cell fate based on variable microenvironmental conditions *in vitro* such as mechanical stiffness, has been described in a 384-well format [21]. This technique enabled observation of changes to cell behavior due to multiple, incremental changes to the biophysical environment, though assay of many changes to the spatial organization and cell compartmentalization of intestinal tissue would not be possible with this model. Spontaneous, gene-targeted, chemical, immune, bacteria, and radiation induced intestinal damage in murine models has provided key insights regarding stiffening/fibrotic initiation or progression and the subsequent impact on epithelial cells [22, 23]. However, translating these results to humans has proven difficult, as mice have comparatively stiffer intestinal tissue than humans, possess a different genotype, and utilize a distinct colonic luminal microenvironment [22, 23]. Murine small intestinal organoids grown on soft (0.6 kPa), physiologic (2.4 kPa), and stiffened (9.6 kPa) scaffolds displayed evaginations of reduced size, decreased Lgr5 expression and increased Olfm4, Muc2 and YAP2 expression as the matrix became less deformable [18]. Thus, stiffer matrices appeared to modulate both epithelial cell stemness and lineage allocation (towards goblet cells) in this model likely through the YAP pathway [18]. A challenge of this work was the use of murine small intestinal organoids which may not entirely mimic human large intestinal disease. Organoids also do not exhibit the clear cell compartmentalization (stem cell niche vs differentiated cell zone) present in the living human intestine. Other recent advances in hydrogel materials properties have enabled comparative studies with organoids on the effect of changing stiffness on intestinal cells but these assays still possess the weaknesses of the organoid model [24, 25]. Human organoids have been placed into matrices of variable stiffness but in this instance only a soft (300 Pa) and physiologic (1.3 kPa) matrix were used [26]. While this work presents evidence that ECM stiffness impacts cell phenotype, no conclusions could be made for excessively stiff matrices. ECM characteristics of *in vitro* intestinal models have also been modulated using decellularized material from a porcine or human intestine to mimic a stiffened mucosa [27–29]. While these models are highly informative and support different Young’s moduli, the standardization of models that use organism-derived ECM components can be challenging due to the required extensive processing with batch to batch variability. Novel, engineered proteins have also been used to tune *in vitro* ECM stiffness, though these studies have focused on mimicking healthy physiology, rather than enabling study of pathophysiologic stiffness [30]. Human embryonic stem cells differentiated into an intestinal lineage have been used to model the fibrotic response in the absence of a change in matrix stiffness by adding inflammatory cytokines [31, 32]. In these studies, no conclusions could be reached, however, regarding the role of matrix stiffness especially given the culture of organoids in very soft Matrigel (Young’s modulus <500 Pa) [33]. While these model systems have revealed valuable insights into intestinal cell physiology, they lack the required features to reveal the impact of a stiff matrix on primary human large-intestinal epithelial cells, particularly the stem cells and their differentiated progeny. An *in vitro* model that mimics the spatial and cellular organization of the human large intestinal crypt with a tunable matrix stiffness would enable an improved understanding of the link between stromal stiffening and human epithelium-related pathology.

We describe a model of intestinal stiffening which integrates primary colonic epithelial cells on a substrate with tunable stiffness. The biophysical attributes of the scaffolding such as the Young’s modulus and diffusion coefficient of various sized molecules was measured. A cross-linked collagen substrate was molded to enable culture of the human epithelial cells on a planar (2D) or 3D crypt-shaped scaffold for observation of cell behavior on physiologic and stiff substrates. Culture on a 2D substrate with or without growth factors was used to mimic the microenvironment of the stem/proliferative cell compartment or the differentiated cell zone in the presence of a physiologic or stiffened stroma. An array of 3D crypts formed on a molded 3D scaffold supported investigation of cell compartmentalization and lineage allocation in the presence of a physiologic or stiffened substrate. The ability of a layer of underlying primary intestinal fibroblasts below the epithelial cells to mitigate impacts of the stiffened surface was also examined. Finally, mRNA gene expression was utilized to assess the differences between cells on a stiff and physiologic substrate. This model builds on prior work utilizing a molded scaffold to create an array of crypts with proper luminal-to-basal polarity and a physiologic stem/proliferative cell niche and differentiated cell zone. Prior work with this model system exclusively employed a scaffold with a stiffness matching that of a healthy individual to recapitulate normal physiology [34]. In the current work, we develop a model with a pathologically stiffened stroma. This new model system supports the culture and assay of primary human, large-intestinal epithelial cells in either a 2D or 3D format within a controlled microenvironment and will serve as a platform for future investigation of epithelial intestinal cells’ response to stiffening as occurs during intestinal disease.

## 2. Results

### 2.1. Development and characterization of a scaffold with a physiologic and an elevated Young’s modulus

**Figure 1.**
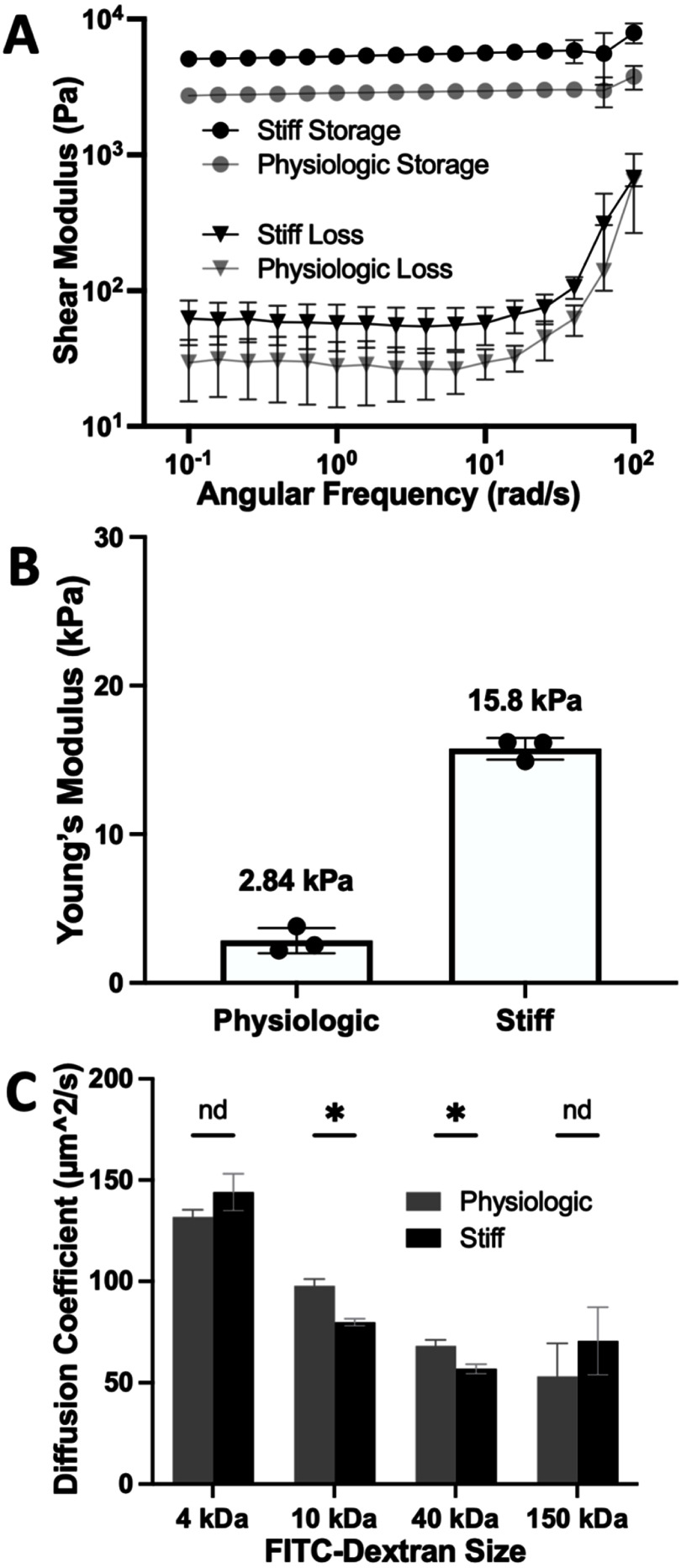
Biomaterials properties of the scaffolds. (A) Rheometric measurement of shear storage and loss moduli (*G*′, *G*′′) as calculated by a frequency sweep with a strain 1% strain amplitude for both physiologic (5.75 mg ml^−1^) and stiff (12.5 mg ml^−1^) collagen hydrogels. (B) Calculated Young’s modulus (*E*′) for physiologic and stiff collagen hydrogels. (C) Measured diffusion coefficient for 4, 10, 40, and 150 kDa fluorescein-conjugated dextran through physiologic and stiff collagen hydrogels. *n* = 3 for all panels, unpaired *t*-tests performed in panel *C*, nd = *p*-value > 0.05, * = *p*-value < 0.05.

Collagen hydrogels (rat-tail, type I) of differing concentrations were used to generate scaffolds of distinct stiffness. Prior investigators have utilized this strategy to modulate the mechanics, structure, and transport within a cell-embedded, or cell-free hydrogel [35–38]. These previous studies demonstrated that, *in vivo* and *in vitro*, increased ECM (such as collagen) concentration leads to increased shear and compressive moduli and increased growth factor loading capacity [35, 37]. Collagen solutions of two different concentrations (5.75 and 12.5 mg ml^−1^) were mixed with cross linkers (N-(3-dimethylaminopropyl)-N ′-ethylcarbodiimide hydrochloride and N-hydroxysuccinimide) and then gelled as a flat 2D scaffold. The collagen concentration and degree of cross-linking were based on prior published data demonstrating that this collagen matrix supports all cell lineages of the normal human large intestine yet can resist cell traction forces preventing scaffold deformation during cell culture [34, 39, 40]. Shear storage and loss moduli of the scaffolds were measured by rheometric testing of collagen hydrogel discs with twisting parallel plates. A cross-linked collagen hydrogel (5.75 mg ml^−1^ collagen) possessed a shear storage and loss moduli of 2842 ± 123 Pa and 29 ± 14 Pa, respectively. In contrast a higher concentration of collagen (cross-linked, 12.5 mg ml^−1^ collagen) yielded a significantly different shear storage of 5287 ± 243 Pa and a shear loss modulus of 58 ± 20 Pa that was not significantly different (*p* < 0.001 for storage modulus and *p* = 0.109 for loss modulus at 0.251–2.51 rad s^−1^), respectively (figure 1(A)). As expected, the cross-linked hydrogel with a higher concentration of collagen was more rigid while the lower concentration was more flexible. The storage moduli (*E*’) were calculated to be 2.84 ± 0.85 kPa (5.75 mg ml^−1^ collagen) and 15.9 ± 0.73 kPa (12.5 mg ml^−1^, figure 1(B)) and determined to be significantly different with an unpaired *t*-test (*p* < 0.001). These values were similar to that published for *ex vivo* measurements of freshly obtained healthy (2.9 kPa) or fibrotic (16.7 kPa) large intestine [3].

The diffusion of molecules through a stiffened matrix can be altered relative to a matrix of physiologic stiffness [38, 41]. Smaller pore sizes in the denser microstructure can act as obstacles for the movement of larger molecules so that increases in the gel collagen concentration can lead to decreased mobility, i.e. diffusion, through a hydrogel [42, 43]. Fluorescence recovery after photobleaching (FRAP) was used to measure the diffusion coefficient (*D*) of various sized fluorescein-dextrans (4–150 kDa) within the physiologic and stiff cross-linked collagen scaffolds. Key intestinal epithelial cell growth factors, Wnt, R-spondin, Noggin (WRN), and EGF possess molecular weights of 40, 35, 26, and 6 kDa respectively, so this range of fluorescein-dextran brackets the size of these key intestinal growth factors [44–47]. No significant difference in *D* was observed for the smallest and largest dextrans in the scaffolds (figure 1(C)). Thus, based solely on *D*, no difference in the concentration profile through the scaffolding is expected for EGF diffusion movement through the two scaffolds. In contrast, both the 10 and 40 kDa dextran possessed a significantly lower *D* in the stiffer compared to the physiologic scaffold. Other investigators have also observed that an increase in collagen concentration impacts the *D* of these intermediate-sized molecules more than that of larger molecules [42, 43]. The difference in *D* was also previously observed to be greatest for molecules with a hydrodynamic radius of 6 nm, approximating the size of a 40 kDa molecule [43, 48].

Differences in the concentration profile of a 40 kDa molecule along the *Z* direction of a scaffold were simulated for the physiologic and stiffened scaffolds (COMSOL Multiphysics). For both scaffolds, the basal and luminal compartment were assumed to act as an infinite source and sink, respectively (due to their much greater volume than that of the hydrogel). At the luminal surface of the flat scaffold, the concentration difference of a 40 kDa molecule was 1.5% greater for the physiologic compared to the stiff scaffold (0.105 nM vs 0.090 nM, supplemental figures S1(A)–(C)) [49]. For cells cultured on the surface of a 3D shaped scaffold, the difference in *D* for a 40 kDa was predicted to yield concentration 1.3, 2.3, and 3.3% greater at the luminal surface, crypt midpoint, and crypt base, respectively for the physiologic versus stiffened scaffolds (supplemental figures S1(E), (F) and supplemental table S3).

Previously measured concentrations for WRN in the basal compartment of both the flat and 3D scaffold were Wnt-3 A (30 ng ml^−1^, ∼1 nM), R-spondin-2 (75 ng ml^−1^, ∼2 nM), and Noggin (71 ng ml^−1^, ∼3 nM) [40]. Reported *K*_D_’s for these growth factors and their receptors are Wnt-3 A (∼10 nM), R-spondin (∼1 nM), and Noggin (∼1 nM) [50–52]. The growth factors are near or below the *K*_D_ for their receptors in the basal reservoir. While this is a concentration regime in which the cells are expected to exhibit the steepest response versus concentration curve, the very small factor differences of 1% for the flat scaffold and <4% for the 3D crypts are unlikely to drive significantly different cellular responses on the physiologic and stiffened scaffolds [53]. In support of this hypothesis is the observation that varying the amount of conditioned medium in the basal compartment between 50%–30% yielded no significant difference in crypt polarization or cell compartmentalization on the 3D scaffolds [54]. Thus, it is unlikely that any observed differences in cell behavior of the two scaffolds would be solely due to differences in the WRN concentration.

### 2.2. Epithelial cell proliferation in the presence of growth factors on a substrate with physiologic or excessive stiffness

**Figure 2.**
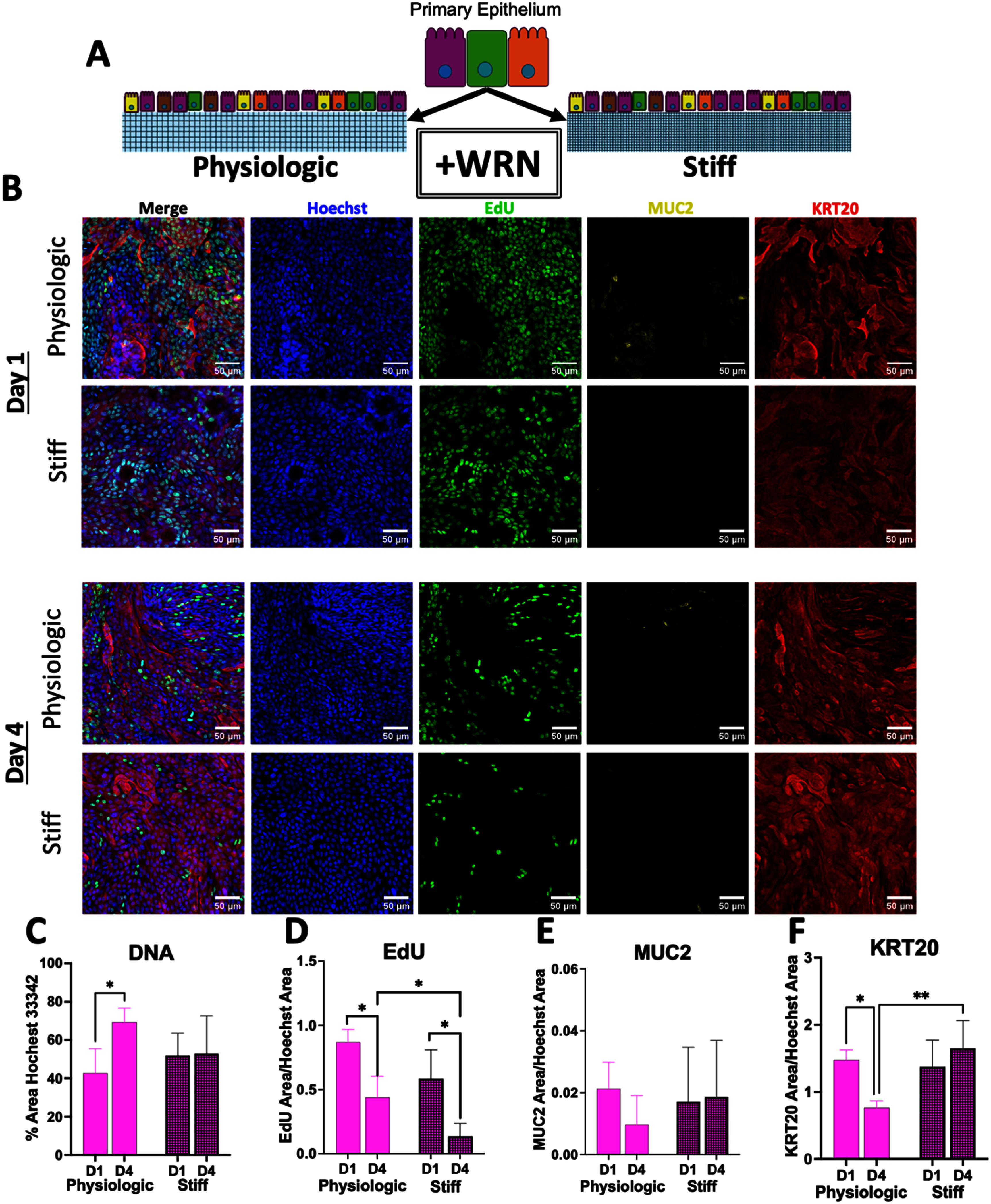
Properties of primary colonic epithelial cells cultured in the presence of growth factors on a flat collagen scaffold of physiologic or elevated stiffness. (A) Schematic depicting culture conditions of epithelium on a physiologic or stiff scaffold while exposed to exogenously provided Wnt, R-spondin, and Noggin (+WRN). (B) Representative fluorescence images (extended focus image (EFI) projection) of epithelium grown on physiologic or stiff scaffolds at day 1 and 4 after the cells became confluent. Staining as follows: Hoechst 33 342 (DNA, blue), S-phase (EdU+, green), mucin 2 (MUC2, yellow), and cytokeratin-20 (KRT20, red). (C) A plot showing the percentage area of the total culture surface positive for Hoechst 33 342 staining above an empirically set threshold. (D–F) Shown is the normalized area positive for EdU-incorporation, Muc2 immunostaining or keratin-20 immunostaining. The normalized area was calculated by dividing the area positive for each of the stains by the Hoechst 33 342+ area. An empirically set threshold was used to determine the area positive for each stain. For panels C–F 3 images (technical replicates) were measured for each of three biological replicates (i.e. *n* = 3). A two-way ANOVA was performed with multiple comparisons conducted by Fisher’s LSD. ns = *p*-value > 0.05, * = *p*-value < 0.05, ** = *p*-value < 0.01.

Primary epithelial cells from the large intestine were cultured on the surface of flat scaffolds with either physiologic or increased stiffness (figure 2(A)). To support proliferative cells, a medium rich in growth factors (WRN) was used for the culture. The cell monolayers were pulsed with EdU 24 h before assay which was on day 1 and 4 after reaching confluency. At the assay time, the cells were fixed and labeled with Hoechst 33 342 (DNA), immunostained for Muc2 (goblet cells) and cytokeratin-20 (KRT20, differentiated epithelial cells), and developed for EdU-incorporation (S-phase cells) (*n* = 3, i.e. three imaged tissue samples for each of three biological replicates). The Hoechst 33 342+ area indicative of total cell number was significantly increased at day 4 on the physiologic scaffold when compared to day 1 (figures 2(B), (C) and supplemental figure S3). In contrast, the Hoechst 33 342+ area showed no significant difference on the stiff scaffold between days 1 and 4. The cells on the physiologic substrate continued to grow in number from day 1–4 whereas those on the stiffer substrate did not. The area positive for EdU incorporation, and Muc2 and KRT20 immunostaining was measured and normalized to the total number of cells by dividing by the Hoechst 33 342+ area. Cells on both substrates possessed a significant decrease in the EdU incorporation, indicating a decreased number of cells in S phase physiologic and stiff scaffolds from day 1 to day 4 suggesting a decreased proliferation rate in both cultures likely due to reaching confluency (figures 2(B) and (D)). However, on day 4, a significantly lower amount of EdU incorporation into cells was present on the stiff relative to the softer scaffolds suggesting that the stiff substrate further decreased proliferation (beyond the contact inhibition present for confluent cells). Very little Muc2 immunostaining was apparent in cells on either substrate and with no statistical difference (*p* < 0.04). The high standard deviation of the data was due to the amount of Muc2 being at or near the limits of detection of the immunoassay. No significant difference was noted across the four days likely due to the high concentration of WRN present inhibiting differentiation (figures 2(B) and (E)). A significant decrease was noted in KRT20 expression from day 1–4 for the physiologic scaffold whereas no significant change was observed for the cells on the stiff substrate (figures 2(B) and (F)). KRT20 primarily marks mature colonocytes and goblet cells but does not label stem cells *i.e.* lgr5+ cells. Thus, the observation that KRT20+ or differentiated cells decreased in number in the proliferating culture under the influence of WRN on the physiologic substrate was consistent with prior observations [55]. More interesting was the unchanged numbers of KRT20+ cells on the stiffened substrate. While KRT20 is largely considered a differentiated cell marker, a minority of proliferative cells can retain KRT20 expression i.e. not a stem cell but not fully differentiated either [56]. The maintenance of KRT20+ cell numbers on the stiff substrate is consistent with the observation of other investigators that intestinal cells on a stiff substrate can enter an intermediate state cell characterized as being no longer a stem cell yet not fully differentiated [18].

### 2.3. Epithelial cell differentiation in the absence of growth factors on a substrate with physiologic or excessive stiffness

**Figure 3.**
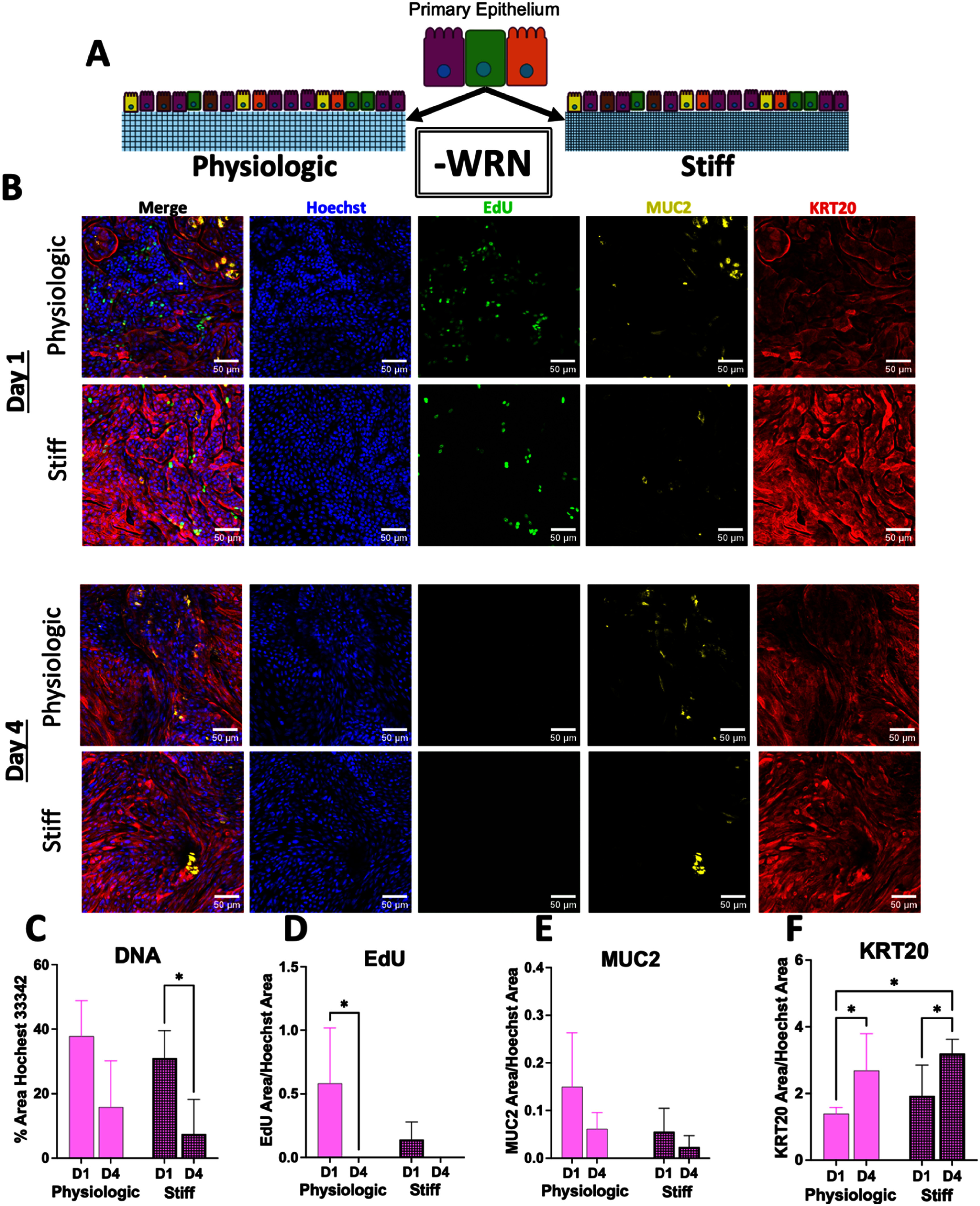
Properties of primary colonic epithelial cells cultured in the absence of growth factors on a flat collagen scaffold of physiologic or elevated stiffness. (A) Schematic depicting culture conditions of epithelium on a physiologic or stiff scaffold without exogenously provided Wnt, R-spondin, and Noggin (-WRN). (B) Representative fluorescence images (extended focus image (EFI) projection) of epithelium grown on physiologic or stiff scaffolds at day 1 and 4 after the cells became confluent while exposed to WRN. Staining as follows: Hoechst 33 342 (DNA, blue), S-phase (EdU+, green), mucin 2 (MUC2, yellow), and cytokeratin-20 (KRT20, red). (C) A plot showing the percentage area of the total culture surface positive for Hoechst 33 342 staining above an empirically set threshold. (D), (E), (F) Shown is the normalized area positive for EdU-incorporation, Muc2 immunostaining or keratin-20 immunostaining. The normalized area was calculated by dividing the area positive for each of the stains by the Hoechst 33 342+ area. An empirically set threshold was used to determine the area positive for each stain. For panels C–F, 3 images (technical replicates) were measured for each of three biological replicates (i.e. *n* = 3). A two-way ANOVA was performed with multiple comparisons conducted by Fisher’s LSD. ns = *p*-value > 0.05, * = *p*-value < 0.05.

Primary epithelial cells from the large intestine were cultured on the surface of flat scaffolds until the monolayer reached confluence (under WRN) after which time WRN was removed to initiate cell differentiation. The cell monolayers were pulsed with EdU 24 h before assay. At the assay (1 and 4 d post confluency) the cells were fixed and labeled with Hoechst 33 342, and stained for Muc2, KRT20 and EdU-incorporation (figure 3(A)) (*n* = 3, i.e. three imaged tissue samples for each of three biological replicates). For cells on both the physiologic and stiff surfaces, large substrate areas free of cells were apparent by day 4 but not present at day 1 (figure 3(B)). However, the measured Hoechst 33 342+ area was only significantly decreased for cultures on the stiff scaffolds at 4 d relative to 1 d (figure 3(C), supplemental figure S4). Since fully differentiated intestinal cells possess a lifetime of ⩽7 d, these cell-free regions were most likely due to the sloughing of dead cells from the surface. On day 1 EdU+ cells were observed on both scaffolds but not in significantly different numbers. By day 4, very few cells on either scaffold were EdU+ consistent with the terminal differentiation of the cells in the absence of growth factors (figures 3(B) and (D)). The normalized area positive for Muc2 was not significantly different for either substrate between days 1 and 4 (figures 3(B) and (E)). On both substrates, the normalized KRT20+ area was significantly increased over time consistent with the differentiation of cells in the absence of growth factors (figures 3(B) and (F)). Remarkably, epithelial cells cultured on the physiologic vs stiff scaffolds displayed few differences (with and without WRN) despite the nearly 6X increase in the scaffold’s Young’s modulus. On both surfaces, the cells actively divided and proliferated in the presence of growth factors and differentiated when growth factors were withdrawn, though decreased proliferation was observed in the presence of growth factors on the stiffened scaffold.

### 2.4. Formation of *in vitro* crypts on a 3D scaffold of physiologic or excessive stiffness

**Figure 4.**
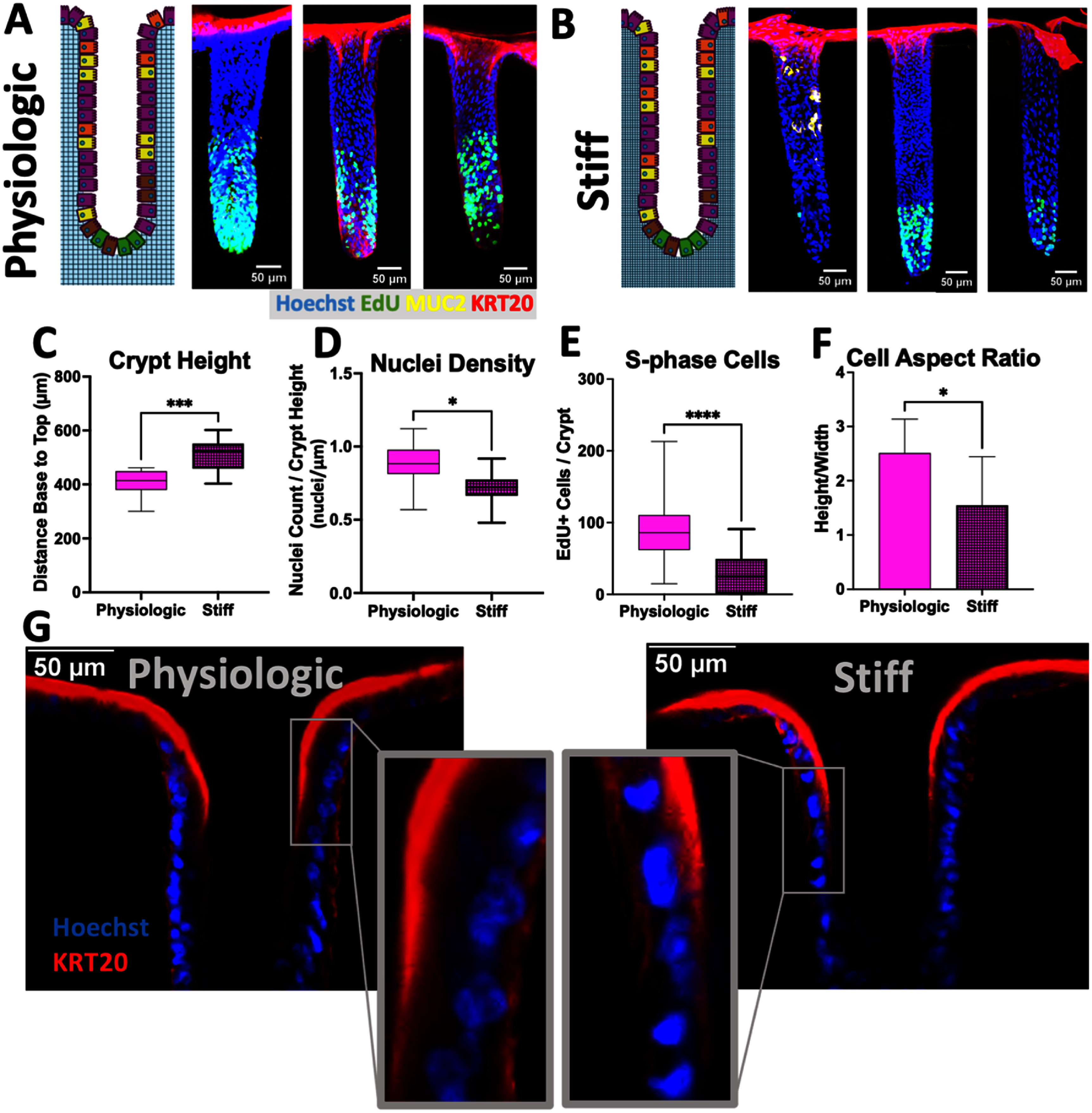
Properties of primary colonic epithelial cells cultured on a shaped 3D collagen scaffold of physiologic or elevated stiffness under a gradient of WRN. (A), (B) Schematic and fluorescence image (maximum *Z*-projection) depicting epithelium cultured on a scaffold of physiologic (A) or increased (B) stiffness. The crypts were fixed, labeled for Hoechst 33 342 binding (blue), EdU incorporation (green) and KRT20 immunostaining (red). (C) Height or length of crypts on scaffolds of physiologic or increased stiffness. (D) Number of nuclei per unit crypt length (nuclei *µ*m^−1^). (E) Average number of EdU+ cells in a single crypt. (F) Cell aspect ratio (height divided by width) for individual differentiated cells within the upper third of crypts. (G) Fluorescence image (single *z*-slice) of the upper one third of a crypt cultured on a scaffold of physiologic or increased stiffness. The crypts were stained with Hoechst 33 342 (blue) and immunostained for EpCAM (red). The central two panels show a selected region at higher magnification. 9 crypts on a physiologic scaffold and 17 crypts on a stiff scaffold were measured for these analyses and unpaired *t*-tests were performed for comparisons in panels C–F. ns = *p*-value > 0.05, * = *p*-value < 0.05, ** = *p*-value < 0.01, *** = *p*-value < 0.001, **** = *p*-value < 0.0001.

*In vivo*, the surface of the intestine is lined with an array of invaginations or crypts in which different cell zones are present. Stem/proliferative cells occupy the crypt base while the short-lived differentiated cells primarily colonocytes (absorptive cells) and goblet cells (producing mucus) occupy the upper crypt regions as well as line the luminal surface. Intestinal cell compartmentalization and movement between the crypt regions *in vivo* is thought to be regulated by microenvironmental gradients (e.g. ECM properties, and differentiation or growth factors) along the crypt length [1]. A gradient in stromal stiffness is also hypothesized to be present along the crypt long axis and how disease states that stiffen this stroma impact the stem cells and lineage allocation is unknown [57]. To assess the impact of scaffolding stiffness on cell compartmentalization and lineage allocation, a molded array of 3D crypts was formed from the physiologic and stiff collagen scaffolds (figures 4(A) and (B)). Primary intestinal epithelial cells were cultured on the scaffolds in a medium high in growth factors (8 d) until a monolayer formed lining the scaffolding surface (including within the invaginations or crypts). Next a gradient of growth factors was applied along the long-axis of the crypts to create a basal stem cell niche rich in WRN and a differentiated cell zone without access to these factors [57–59]. After 4 d under the growth factor gradient, cells were pulsed with EdU and then on day 5, the cultures were fixed and labeled with Hoechst 33 342, immunostained for KRT20 and stained for EdU incorporation. Crypts on the stiffer matrix were significantly longer than those on the softer matrix (figure 4(C)). The molded crypt length prior to cell culture was 500 *µ*m for both scaffolds and the measured crypt length after cell culture for 9 d on the scaffold was 510 ± 56 *µ*m for the stiff crypts (*n* = 9) vs 408 ± 51 *µ*m for crypts on the physiologic scaffold (*n* = 17), (figure 4(C)). Thus, the stiff scaffolding retained the original molded shape more accurately than the softer scaffold which was likely deformed by traction forces from the cells [36]. On both scaffolds, a clear basal stem cell compartment and luminal differentiated cell region was formed in the majority of crypts (figures 4(A) and (B)). However, significantly more crypts were populated with at least one EdU+ cell for the physiologic relative to the stiff scaffold (2.8% vs 0.87% of total area, supplemental figures S2(A), (B)). Of the crypts that did possess EdU+ cells, there were significantly more cells, and EdU+ cells/crypt on the physiologic compared to the stiff surfaces (figures 4(D) and (E)). A differentiated cell compartment (KRT20+ region) was present along the upper crypt region as well as along the luminal surface for both scaffolds. However, the cells on the physiologic scaffold possessed a significantly greater aspect (height to width) ratio than the cells on the stiff scaffold (figures 4(F) and (G)). The measured aspect ratio *in vivo* is 3–5 (35 *µ*m height, 9 *µ*m width *in vivo*) [34, 60]. The cell morphology on the softer surface more closely resembled the *in vivo* differentiated columnar epithelium than the cells on the stiffer scaffold. The columnar-shaped cells also accommodate a significantly greater number of nuclei per length of crypt relative to the more cuboidal cells on the stiffer surface (figure 4(D)). A potentially confounding variable in these measurements is the different lengths of the crypts on the physiologic and stiff scaffold due to cell forces (figure 4(C)). However, the distance between the porous membrane (or basal reservoir with WRN) and the crypt base was not significantly different between the two scaffolds (64 ± 18 *µ*m, physiologic vs 55 ± 15 *µ*m, stiff, supplemental figures S1(G) and (H)). The different crypt lengths were then computationally modeled to assess whether the cells in the crypt base or top surface might be exposed to substantially different growth factor gradients (supplemental figure S2). At the crypt base, the model predicted a 1.5% higher concentration for a 40 kDa molecule in a stiff crypt relative to that of a crypt on a scaffold of physiologic stiffness. Overall, the computational model suggests that the concentration of growth factors at the crypt base was not likely the source of the epithelial cell differences between a stiffened and physiologic scaffold. From these data it appears that a stiffened scaffold altered the morphology and proliferation behavior of intestinal epithelium within *in vitro* 3D crypts.

### 2.5. Impact of fibroblasts cocultured with epithelial cells on 3D scaffold of excessive stiffness

**Figure 5.**
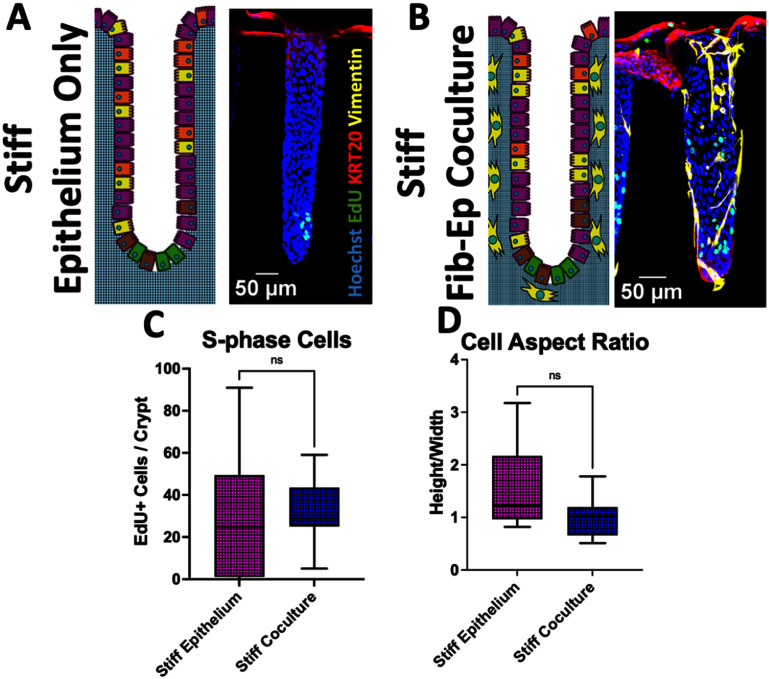
Properties of primary colonic epithelial cells cultured on a shaped 3D collagen scaffold of elevated stiffness with or without intestinal fibroblasts. (A), (B) Schematics and maximum *Z*-projection fluorescence images of 3D crypts with stiffened underlying scaffolds containing (A) epithelium alone or (B) epithelium cultured above fibroblasts. The crypts were fixed and labeled for Hoechst 33 342 binding (blue), EdU incorporation (green), KRT20 immunostaining (red), and vimentin immunostaining (yellow, fibroblasts). (C) Plot displaying the number of proliferative cells identified in individual crypts. (D) Plot displaying the cell aspect ratio (height divided by width) for individual cells within crypts. Epithelium only (17) and coculture (29) crypts were measured for these analyses and unpaired *t*-tests were performed for comparisons in panels C and D. ns = *p*-value > 0.05, * = *p*-value < 0.05.

 Fibroblasts play an integral role in supporting epithelial cell proliferation and differentiation *in vivo* [1, 61]. Fibroblasts also provide soluble growth factors and deposit ECM for epithelial cell maintenance, and prior studies demonstrated that fibroblasts could modulate epithelial proliferation and support barrier function *in vitro* [62–66]. To determine whether an underlying layer of fibroblasts might mitigate the impact of a stiffened scaffolding on the epithelial cells, crypt arrays were cultured with a layer of fibroblasts underlying the epithelial cells (figures 5(A) and (B)) (n = 17 epithelium only and n = 29 coculture crypts). The number of proliferative cells in coculture versus epithelial monoculture was not significantly different with and without the underlying fibroblasts (figure 5(C)). No significant difference was observed in cell shape (aspect ratio) when fibroblasts were present relative cells in crypts without fibroblasts. Under these conditions fibroblasts did not mitigate the impacts of the stiffened substrate on the epithelial cells within *in vitro* crypts (figure 5(D)). *In vivo*, mesenchymal cells underlie the epithelial cells and are present throughout the lamina propria. Under these conditions, the proportion of fibroblasts to epithelial cells may not have been sufficient to ‘rescue’ the epithelial cells. Alternatively, once stromal stiffening occurs, fibroblasts alone (in any ratio to the epithelial cells) may not be able to undo impacts on the epithelial cells.

### 2.6. Impact of a physiologic and stiffened scaffold on gene expression

**Figure 6.**
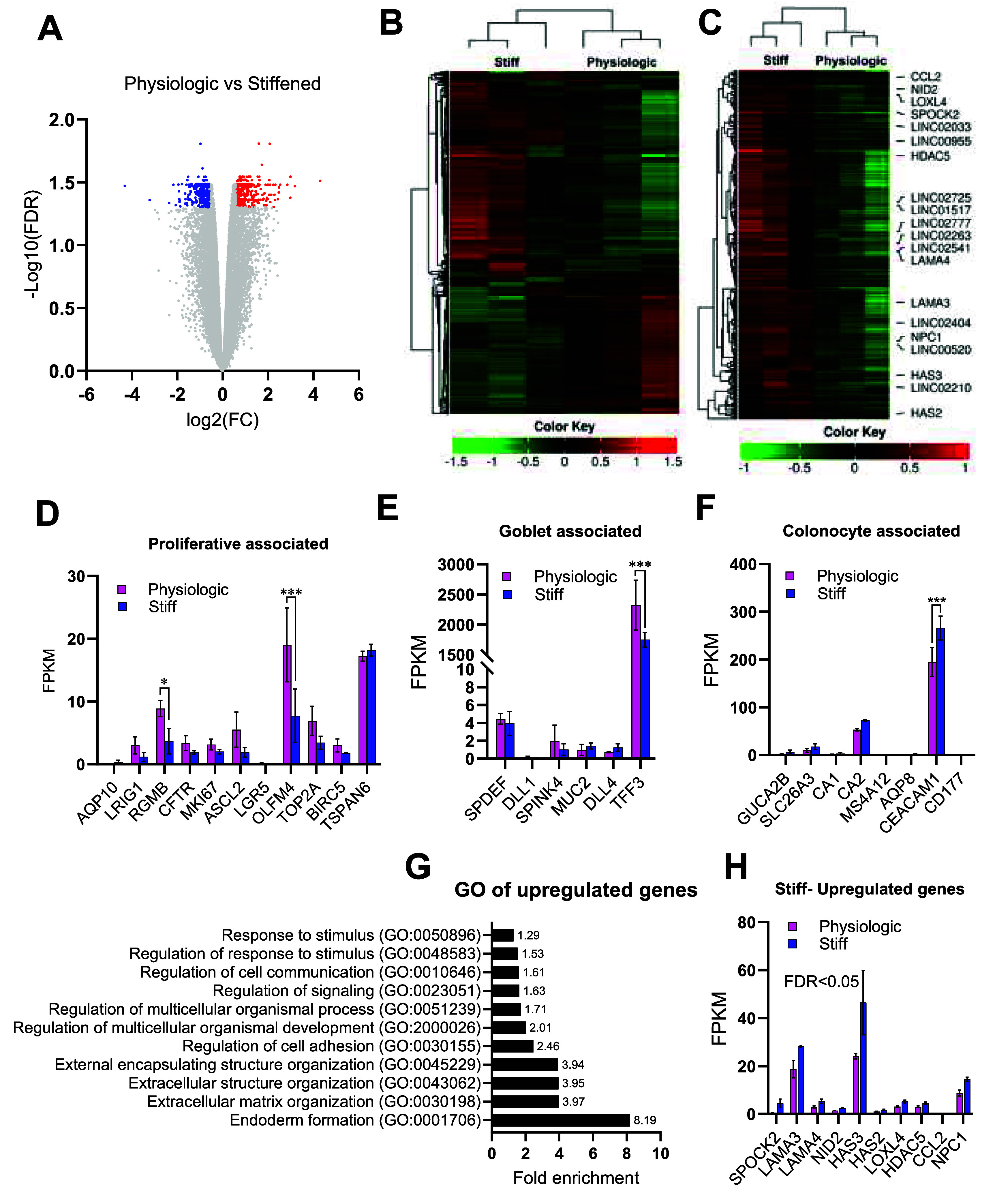
Bulk RNA-sequencing cells from 3D crypt arrays on physiologic and stiff scaffolds. (A) Volcano plot comparing the false discovery rate (-log10 (FDR)) versus the fold change (log2 (FC)) in gene expression for cells cultured on physiologic versus stiff scaffolds. Transcripts identified in red indicate significantly upregulated genes (378 total), and in significantly downregulated genes (385 total). (B) Heatmap with hierarchical clustering ranking the top 2000 upregulated genes using the Pearson method for distance and average linkage across 3 cell samples cultured on stiffened (left) or physiological scaffolds (right). (C) Heatmap biclustered for the 378 up-regulated genes in cells on stiffened vs physiological scaffolds. A row dendrogram for the genes is shown on the left *Y*-axis and gene labels on right *Y*-axis while the columns represent the different samples (D) per million fragments mapped (FPKM) is shown for various genes associated with proliferative cells and differentially expressed on cells grown on stiff vs physiologic. FPKM was normalized using trimmed mean of M values (TMM) normalization. (E) Normalized FPKM for goblet cell-associated genes. (F) Normalized FPKM for enterocyte-associated genes. (G) Over-represented cell functions in up-regulated genes in cells on a stiffened compared to a physiologic scaffold obtained by ontology gen (OG) analysis. (H) Up-regulated genes in cells on a stiffened scaffold (compared to a physiologic) associated with over-represented cell functions (FC > 2) identified in OG analysis. Analyzed by two-way ANOVA with Tukey’s multiple comparisons test. Significative difference, *P < 0.05, ***P < 0.0001.

To gain insight into the mechanisms operating to increase proliferation on the physiologic relative to the stiff 3D scaffolds, bulk RNA was isolated from whole crypt arrays formed on both types of scaffolds. mRNA for 19 837 unique RNA transcripts was detected and compared (figure 6(A)). Of these transcripts, 385 were significantly downregulated, and 378 were significantly upregulated on stiff compared to physiologic scaffolds (figure 6(A), supplemental table S2). Heat maps were created to illustrate the pattern in differential gene expression for the RNA-seq data set (figure 6(B)) as well as for the significantly upregulated genes (figure 6(C)). Genes with similar expression pattern and higher expression levels are highlighted (0–1.5 red scale) in the upper part of three first columns and were most prevalent on the stiffened substrate while the lower clusters of genes (−1.5–0 green scale) for genes with lower expression levels compared with physiological samples. These data suggest that cells on a stiffened vs physiologic substrate displayed distinct groupings of gene expression. The samples clustering confirmed similarity in gene patterns associated with the substrate. Many markers of proliferative/progenitor cells demonstrated a significant increase in expression levels in cells on the physiologic vs stiff scaffold consistent with the increased amount of EdU incorporation in cells on physiologic vs stiff scaffolds (figure 6(D)). Next genes expressed predominantly in differentiated cells were examined initially. Most goblet cell and colonocyte-associated genes were expressed at similar levels (figures 6(E) and (F)). These data suggested that these mature nondividing cells may be in similar states in the two cultures, consistent with the prior data.

A GO Analysis (geneontology.org) was conducted on the upregulated genes (table S4) to determine whether common biological processes might be identified (figures 6(G) and (H)). The expression of multiple ECM proteins or ECM modifying proteins was significantly altered between the two cultures. Laminins are cell adhesion proteins acting as connectors between cells and the basement membrane. Both laminin A subunits 3 and 4 (LAMA3, LAMA4), were upregulated in the stiff versus the physiologic substrates consistent with the known role of laminins in resisting tensile forces which are increased in a stiffened substrate [67]. Laminin A directly binds to Nidogen-2 (NID2), a cell adhesion protein of the basement membrane and up-regulation of NID2 was also present in epithelial cells cultured on the stiffened relative to physiologic substrates (*P* = 0.000085). The matrix synthetic and modifying enzymes hyaluronan synthase 2 and 3 (HAS2, HAS3) and lysyl oxidase-like 4 enzyme (LOXL4) demonstrated significantly increased expression in the stiffened substrates relative to the physiologic scaffold (*p*= 0.00127 for HAS2, *p* = 0.00037 for HAS3, *p*= 0.000226 for LOXL4). HAS2/3 are required for the synthesis of hyaluronic acid (HA), a major component of the ECM while lysyl oxidase-like 4 (LOXL4) enzymes catalyze the cross-linking of collagen and elastin. Increased deposition of HA and collagen cross-linking are associated with a stiffened ECM and enhanced tumorigenesis. Significantly more SPOCK2 or testican-2 mRNA was present in cells cultured on a stiff relative to a physiologic scaffold. SPOCK2 encodes an ECM protein often misregulated in tumor cells [68]. These data suggest that cells on the stiffened substrate actively responded to and attempted to modify the properties of their underlying substrate.

Interestingly proteins regulating nuclear function were significantly altered between the physiologic and stiff substrates. Ten different long intergenic non-protein coding RNAs (LINC00955, LINC02033, LINC02725, LINC00520, LINC02404, LINC01517, LINC02777, LINC02263, LINC02541, LINC02210) were upregulated in the cells on stiff versus physiologic substrates. LINC complexes induce stretching of nuclear pore complexes decreasing pore mechanical resistance and increasing translocation of proteins into the nucleus. The LINC and NPC proteins can enhance translocation of proteins such as YAP/TAZ, protein sensors of mechanical cues from the ECM and that translocate into the nucleus in response to stiffened substrates. Histone deacetylase 5 (HDAC5) which regulates histone function in the nucleus was up-regulated (*P* = 0.000 85) in cells on stiffened compared to physiologic substrates and increases in HDAC5 are linked to tumorigenic behavior [69]. Cells on the stiffened substrates, not surprisingly modulate nuclear function, in order to adapt to their microenvironment. mRNA expression for proteins involved in response to cellular stress was also observed to be significantly increased in the cells on stiff versus physiologic scaffolds. For example, CCL2 (C–C motif chemokine ligand 2), also known as monocyte chemoattractant protein-1 (MCP-1) was significantly upregulated (*P* = 0.00033) in the cells on stiff surfaces. CCL2 is an immune cell attractant thought to play a role in autoimmune intestinal disease and fibrotic responses.

Niemann–Pick disease type C1 (NPC1) was upregulated in cells on the stiff versus physiologic scaffold (*p* = 0.000122). This transcript encodes for a transporter that moves cholesterol and fatty acids out of endosomes and lysosomes and its upregulation is often observed in tumors, likely due to increased synthetic demands. Transmembrane protein 109 (TMEM109) which encodes a voltage-gated cation channel in the endoplasmic reticulum was downregulated in cells exposed to stiff vs physiologic substrates (*p* = 0.00026). TMEM109 is known to be modulated in the presence of a variety of stressors including a stiffened substrate [70].

## 3. Conclusions

Stromal stiffening in the intestine due to excessive ECM deposition is a component of many intestinal diseases *in vivo*. To investigate how intestinal stiffening impacts epithelial cell behavior, a flat and 3D-crypt shaped *in vitro* model was created to mimic the biophysical characteristics of healthy and stiffened intestinal tissue. Rheometric testing revealed that these two substrates, containing 5.75 mg ml^−1^ and 12.5 mg ml^−1^ collagen, exhibited shear and compressive moduli that are similar to healthy and stiffened colonic tissue, respectively. Experimentally determined diffusion coefficients within these two scaffolds were significantly different for EGF and Wnt-sized molecules, though finite element analysis suggests that these differences in diffusion have a minimal impact on the concentration of these growth factors in the crypts made from a physiologic or stiffened scaffold under these conditions. Primary human colonic epithelium was cultured on the scaffolds to form an *in vitro* model of a physiologic and stiffened intestinal stroma. On a planar substrate, epithelial cell proliferation, differentiation, and nuclei density was altered on the surface of stiff scaffolds when compared to the substrate of physiologic stiffness. In the 3D crypts, proliferative and differentiated cell zones were formed on both scaffolds although epithelial cell proliferation was diminished in the stiffer relative to physiologic scaffolds. Measurements of gene expression revealed that a stiffened scaffold elicits altered gene expression within epithelium as well. In accordance with prior observations, many ECM related genes were upregulated in the stiffer context (e.g. LAMA, HAS, LOXL, SPOCK). Investigations using murine small intestinal organoids previously indicated that cells with the proliferative cell marker OLFM4 were increased in number on a stiff relative to physiologic substrate [18]. In this study we observed downregulation of OLFM4 in the bulk tissue on a stiff scaffold, thus highlighting the need for further spatial investigation and perhaps showing a nuanced difference between murine vs human or large vs small intestine gene expression in response to a stiffened matrix [71]. In the future, the chemical microenvironment (in addition to the biophysical microenvironment) might be incorporated to develop a fibrosis model, for example, by addition of inflammatory factors such as transforming growth factor beta or tumor necrosis factor alpha. This microphysiologic system, however, recapitulates multiple aspects of the biophysical changes observed with intestinal stiffening all while offering precise control of the culture conditions, use of cells from different individuals, incorporation of multiple cell types and compatibility with multiple downstream assays. This organ-on-chip mimicking key colonic architectural features and cell compartmentalization offers a tool to begin to dissect intestinal epithelial cell responses to a stiffened stroma as occurs in many disease states.

## 4. Materials & methods

### 4.1. Measuring hydrogel moduli

The hydrogels were cast by mixing N-(3-dimethylaminopropyl)-N ′-ethylcarbodiimide hydrochloride (EDC, 0.6 M (Oakwood Chemical, cat. no. 25 952-53-8)) and N-hydroxysuccinimide (NHS, 0.15 M (Millipore-Sigma, cat. no. 130672)) with rat tail type I collagen (Corning, cat. no. 354236) which had been lyophilized and then dissolved in MES buffer (Millipore-Sigma, cat. no. M8250, 0.1 M, pH 5) at a ratio of 1:1:8 by volume, as described by Hinman *et al* [58]. The collagen concentration prior to crosslinking with EDC and NHS was at a concentration of 5.75 mg ml^−1^ or 12.5 mg ml^−1^. Prior to rheometric measurements, the hydrogel was cast between two glass slides covered by parafilm with 1 mm spacer. The resultant 1 mm tall collagen hydrogel slabs were cut into 8 mm diameter discs. The moduli were measured on an Anton Paar MCR-301 Rheometer (Anton Paar USA, Ashland VA) equipped with an 8 mm-diameter parallel-plate platen. The gap was set at 0.8 mm and a frequency sweep was conducted between 0.1 and 100 rad s^−1^ with 1% strain amplitude. The shear storage and loss moduli were calculated from the linear region of the frequency sweep from 0.251–2.51 rad s^−1^ for each of three replicates (*n* = 3). From the shear storage modulus (G’) the young’s modulus (E’) was calculated as (E’ = 2 G’(1 + v)) assuming a Poisson Ratio (v) for the hydrogel of 0.49 [72].

### 4.2. Diffusion measurement with FRAP

For diffusion measurements, thin hydrogels (0.3 mm thick) were cast between two glass slides covered with parafilm. For each test, three hydrogel discs (*n* = 3) with a 3 mm diameter were formed using a biopsy punch, and the samples were placed into a solution containing 100 ng ml of various sized FITC-conjugated dextran (4, 10, 40, and 150 kDa) and the samples incubated for 2 h to enable equilibration with the dextrans. Next the dextran-loaded hydrogels were placed on the stage of a confocal microscope (Olympus Fluoview 3000 equipped with 405, 488, 561, and 640 nm laser diodes). With a 20x objective (NA = 0.45) at 5x digital zoom, a small circular area was photobleached with the 405 nm laser at the maximum allowable laser power for 1 min. Immediately following photobleaching, the same objective at 1x zoom was used to obtain images every 1.1 s for 100 frames with the 405 nm laser at a setting of ‘2%’ of maximal power. A fluorescence intensity profile (over distance) at time 0 s was used to determine the effective radius (*r*_e_) of the bleached area. The fluorescence intensity recovery curve over time was then used to determine the half-time of recovery (*Tau*) and the nominal radius (*r*_n_) was set as 75 *µ*m for each test [73, 74]. From these the diffusion coefficient was determined by the following equation:

\begin{equation*}{D_{{\text{confocal}}}} = \frac{{{r_{\text{e}}}^2 + {r_{\text{n}}}^{\text{2}}}}{{8{\tau _{1/2}}}}.\end{equation*}

### 4.3. Computational modeling of concentration profiles in 3D crypts

COMSOL Multiphysics (COMSOL Inc., v. 5.5, www.comsol.com. Burlington, MA) was used to model the concentration gradient of a 40 kDa molecule through the collagen hydrogel scaffold. For both scaffolds, the basal and luminal compartment were assumed to act as an infinite source and sink (due to their much greater volume than that of the hydrogel). No flux was permitted through the sides of the hydrogel, and the luminal and basal reservoirs containing media were modeled as water. The geometry of the flat collagen scaffolds or the 3D crypt scaffolds in a 2D simulation was modeled using the transport of diluted species module. The diffusion coefficients determined experimentally for a 40 kDa-dextran in the different scaffolds was used i.e. for the physiologic scaffold, 6.824 × 10^−11^ m^2^ s^−1^ and the stiff scaffold, 5.681 × 10^−11^ m^2^ s^−1^. An experimentally measured source concentration of 1 nM (30 ng ml^−1^) was used, and the static state equilibrium was computed in a course mesh. In both flat and crypt scaffolds, concentration along a vertical line was plotted to show the predicted concentration of a 40 kDa (Wnt-sized) molecule at physiologically relevant points throughout the simulation.

### 4.4. Cassette construction and scaffold casting

Collagen hydrogel scaffolds were placed within a modified hanging basket as described previously [58]. In brief, the factory-supplied membrane on the base of a 12-well hanging basket (Corning, cat. no. 354236) was removed and replaced with a permeable membrane (Millipore-Sigma, cat. no. BGCM00010) and then an impermeable membrane (McMaster-Carr, cat. no. 8689K44) with a 3 mm diffusion window was placed on the basal side of the permeable membrane. Hydrogel scaffolds were cast as described above (mixing EDC/NHS/rat tail type 1 collagen), but the luminal surface was shaped by molding against a flat surface comprised of poly(dimethyl siloxane) (PDMS) or the PDMS negative of crypt-shaped microwells. The flat scaffolds were formed from 50 *µ*l of EDC/NHS/collagen loaded into the luminal compartment of the modified hanging basket. For the 3D-crypt microstructures a PDMS stamp (80 *µ*m wide, 500 *µ*m tall posts) was placed in to from 50 *µ*l of EDC/NHS/collagen loaded into the luminal reservoir of the modified hanging basket. The molds were maintained on the collagen for 1 h under pressure (25 psi) to enable the collagen to gel and crosslink with minimal bubbles. The PDMS molds were fabricated as described previously [58]. After removal of the molds from the collagen surface, the collagen was incubated in deionized water over night (2 l per 12 inserts) to remove excess cross-linking reagents. The constructs were cleaned by incubation in 75% ethanol for 5 min. The scaffolds were then rinsed with PBS three times. A solution of human type I collagen (10 ng ml^−1^ VitroCol in PBS, Advanced BioMatrix, cat. no. 5007-20ML) was added to the luminal compartment incubated for 12 h to place a thin layer of human collagen across the scaffolds.

### 4.5. Cell culture

Human colon epithelial cells from a transverse colon tissue sample (male, 23 years old, RRID: CVCL_ZR41) were expanded and maintained on a flat, non-crosslinked collagen slab as described previously [55]. Primary fibroblasts isolated from descending colon (male, 12 years old, RRID: CVCL_D6WE) were expanded and maintained within a culture flask stored horizontally with Dulbecco’s Modified Eagle Medium (DMEM, Thermo Fisher, cat. no. 11995065) supplemented with 10% heat-inactivated fetal bovine serum, 100 U ml^−1^ penicillin, and 100 *μ*g ml^−1^ streptomycin (table 2.1).

For seeding within molded scaffolds, fibroblasts were added to the luminal side of well-inserts in 1 mL DMEM at a concentration of 3 × 10^5^–5 × 10^5^ cells ml^−1^ for both planar and 3D crypt scaffolds. Then medium was replenished daily. Epithelial cells were isolated and dissociated as described previously using collagenase type 4 (Worthington Biochemical Corporation, cat. no. LS004189) and TrypLE express enzyme (Thermo Fisher, cat. no. 12605028) [58]. Epithelial cells were then added directly to the scaffolds for culture. In 3D crypts, cells were grown in EM (supplemental table S1) for 8 d (replenished daily) and then polarization was executed by replacing basal medium with stem medium (SM, supplemental table S1) and luminal medium with differentiation medium (DM, supplemental table S1). After 5 d of polarization (media replenished daily), the samples were fixed and stained for endpoint analysis.

### 4.6. Fluorescence staining and confocal imaging

EdU (5-ethynyl-2’-deoxyuridine, 1 *µ*g ml^−1^) was added to cells 24 h before fixation. Cells were fixed with Prefer fixative (Anatech Ltd, cat. no.NC9053360) for 20 min, permeabilized with 0.5% Triton X-100 at 20 °C, and then blocked with 1% bovine serum albumin (BSA) for 1 h. Subsequently, integrated EdU was stained with sulfo-Cy5-azide (1.25 *µ*g ml^−1^) and then primary antibodies were added [75]. In this study, a DNA stain (Hoechst 33342, Sigma-Aldrich, cat. no. B2261) and antibodies for mucin-2 (MUC2, Santa Cruz, cat. no. sc-15334), cytokeratin-20 (KRT20, Cell Signaling Technology, cat. no. 13063S), and vimentin (VIM, Santa Cruz, cat. no. sc-6260) were used (all 1:500 dilution). After overnight incubation at 4 °C with primary antibodies, the samples were rinsed and matched secondary antibodies labeled with Alexa Fluor 488 (goat anti-mouse, Life Technologies Corp., cat. no. A28175), and Alexa Fluor 555 (donkey anti-rabbit, Life Technologies Corp., cat. no. A31572) were added and the samples incubated for 12 h at 4 °C. Confocal microscopy was performed using an Olympus Fluoview 3000 epifluorescence microscope equipped with 405, 488, 561, and 640 nm laser diodes and 4x, 10x, or 20x magnifying objectives to obtain images. Detected emission spectra used were Hoechst 33342: 430–470 nm, Cy5: 650–750 nm, AF 488: 505–545 nm, AF 555: 560–580 nm.

Cell Profiler (cellprofiler.org [76]) was used to segment and measure the area positive for each fluorescent marker (above an empirically set threshold). Monolayers were imaged with a 10x objective, and a maximum intensity projection of the *z*-stack (step size 4 *µ*m) was assessed. For 3D crypts, images (4x objective, N.A. 0.16, *z*-stack with a slice depth of 25.4 *µ*m) and a maximum intensity *z*-projection was used to analyze EdU positive area on the full crypt arrays. Next, a pair of microdissection scissors was used to bisect the crypt array so that the array could be imaged from the side as described previously [54]. A 20x objective (N.A. = 0.45, *Z*-slice depth of 4 *µ*m) was used image the crypts and then *z*-stacks were reconstructed in 3D using IMARIS X64 v9.8.2 (imaris.oxinst.com, Oxford Instruments). The number and position of nuclei (Hoechst 33342+), and proliferative nuclei (EdU+) were counted using the IMARIS spots module and these data were used to calculate values like crypt height, nuclei density, and proliferative cell count. Additionally, CellSens (Olympus, Fluoview FV3000, Waltham, MA) was used to measure the height and width of individual differentiated cells in the upper half of crypts visualized with KRT20 staining and these data were used to calculate individual cell aspect ratio.

### 4.7. Bulk RNA-sequencing

To access differences in relative gene expression, RNA was separated from 3D crypt arrays containing epithelial cells only. This genetic material was extracted from three physiologic stiffness scaffolds and three stiffened scaffolds, and the contents was taken from a cut section of tissue taken from above the 3 mm diffusion window found in the modified cell culture inserts. Cell arrays were agitated via vortexing within RNA lysis buffer (Zymo Research, cat. no. R1057,) and RNA was then extracted with a Quick-RNA™ MiniPrep Plus kit (Zymo Research, cat. no. R1057). Sequencing was then conducted on an Illumina NextSeq 2000 (Illumina. San Diego, CA). Cutadapt was applied to remove adapter sequences. STAR with 2-pass mapping was used to align reads to the reference genome and gene annotation along with quantification of gene-level expression. FastQC, RNA-SeQC, RSeQC were used to check various QC metrics including insert fragment size, read quality, read duplication rates, rRNA rates, gene body coverage and read distribution in different genomic regions.

### 4.8. Analysis of differentially expressed genes

The Bioconductor package, edgeR3.36.0, was used to detect differential gene expression between sample groups [77]. Genes with low expression were excluded using an edgeR function filterByExpr with min.count = 10 and min.total.count = 15. The filtered expression matrix was normalized using the trimmed mean of *M*-values (TMM) method. Differential gene expression was considered significant if absolute log2 fold change was above 1 (i.e. fold change > 2 in either direction), and Benjamini–Hochberg adjusted *p*-values were less than 0.05. A volcano plot was used to graph the differential gene expression in the RNA-seq data set and visualize changes in gene expression between stiffened and physiologic conditions. GraphPad PRISM 9 software (version 9.5.0) was used to generate the volcano plot.

### 4.9. Hierarchical gene expression clustering

Categorization of the top 2000 genes was performed online using integrated differential expression and pathway analysis (iDEP) [78]. The subset of genes was ranked by distribution variances using ANOVA analysis of total read counts across sample groups. Another subset of 378 significantly up-regulated genes was also hierarchically patterned. The expression values (fragments per kilobase of exon model per million mapped, FPKM) from RNA-seq data of six samples (3 stiffened and 3 physiological) were clustered using log2-transformed data by the Pearson correlation method [78]. The average linkage clustering method was used to determine the distance between clusters to create the heatmaps with groupings of genes between the stiffened and physiological samples.

### 4.10. Over-representation enrichment analysis

An over-representation test was performed on upregulated genes in the Homo sapiens database using PANTHER v.16.0 (Protein Analysis THrough Evolutionary Relationship, released 2024-08-07), with Fisher’s exact test and FDR correction for character vectors [79]. Fold-enrichment values for all genes were used in pathway analysis by Gene Set Enrichment Analysis, and the gene ontology (GO) functional categorizations were downloaded from the GO database (DOI: 10.5281/zenodo.14861039, released on 2025-02-06).

### 4.11. Statistics

Data are presented as mean ± standard deviation on all plots. Differences between means from separate groups were determined using student’s *t*-tests, unless otherwise specified. Statistical analysis and graphical illustration were performed using GraphPad PRISM 9 software, version 9.5.0 (GraphPad Software, San Diego, CA) at a significance level (*α*) of 0.05 unless otherwise noted.

## Data Availability

The data that support the findings of this study will be openly available following an embargo at the following URL/DOI: http://doi.org/10.5061/dryad.fxpnvx15v [80]. Supplementary Data 1 available at http://doi.org/10.1088/1758-5090/ae2cf2/data1. Supplementary Data 2 available at http://doi.org/10.1088/1758-5090/ae2cf2/data2. Supplementary Data 3 available at http://doi.org/10.1088/1758-5090/ae2cf2/data3.
